# Caffeine Expectancy Does Not Affect Interval Running Performance, Physiological Responses, or Running Kinematics in Trained Runners: A Randomized Controlled Crossover Trial

**DOI:** 10.3390/nu18152492

**Published:** 2026-08-02

**Authors:** Fernando Valero, Fernando González-Mohíno, Alejandro Alda-Blanco, Sergio Rodríguez-Barbero, Violeta Muñoz de la Cruz, Daniel Juárez Santos-García, Juan José Salinero

**Affiliations:** 1 Sport Training Lab, Faculty of Sport Sciences, University of Castilla-La Mancha, 45071 Toledo, Spain; fernando.valero@uclm.es (F.V.); fernando.gmayoralas@uclm.es (F.G.-M.); alejandro.alda@uclm.es (A.A.-B.); sergio.rexposito@uclm.es (S.R.-B.); violeta.munoz@uclm.es (V.M.d.l.C.); daniel.juarez@uclm.es (D.J.S.-G.); 2 Facultad Ciencias de la Salud y Escuela de Doctorado, Universidad Internacional de La Rioja, 26004 Logroño, Spain

**Keywords:** placebo induced, interval training, endurance, perceived exertion

## Abstract

**Objectives**: This study aimed to analyze the placebo effect of caffeine expectancy on interval running performance in moderately trained athletes. **Methods**: Participants were randomly allocated to one of two treatment sequences (placebo–control or control–placebo) and completed two 5 × 1000-m training sessions on an outdoor athletics track under two conditions: (1) ingestion of a placebo capsule described as containing caffeine (3 mg·kg^−1^), and (2) a control condition (no ingestion). Running performance (primary outcome), pacing strategy, heart rate, kinematic variables, perceived exertion (RPE), and side effects were recorded. **Results**: Fourteen participants were randomized and all completed both study periods and were included in the analyses. No statistically significant differences were observed in total session time between conditions (1021.50 ± 114.01 vs. 1017.23 ± 109.08 s, control vs. placebo, respectively; *p* = 0.270; ES = 0.31). Likewise, no differences were found in pacing strategy, heart rate, rating of perceived exertion, or running kinematics (all *p* > 0.05). However, participants reported greater feelings of activeness and higher ratings of selected caffeine-related side effects, including increased urine production and sleep disturbances, under the placebo condition. **Conclusions**: Believing that caffeine had been consumed did not improve interval-running performance, pacing strategy, physiological responses, or running kinematics in trained runners. However, caffeine expectancy altered the perception of some expected caffeine-related side effects. These findings provide limited evidence for an ergogenic placebo effect of caffeine during interval training and highlight the need for further research to identify the conditions under which expectancy effects may influence exercise performance.

## 1. Introduction

The placebo effect is a positive outcome resulting from the belief of having received a beneficial treatment or condition, such as the belief that one has ingested caffeine to enhance sports performance [1]. Recently, following a consensus statement developed by different authors on the scope of the placebo effect in sport [2], it has been defined as a desirable outcome derived from personal expectations and/or learned responses to a treatment, a situation, or an inert substance that leads athletes to believe they are using an ergogenic aid to achieve improved performance through an enhancement of their psychological perception [3]. Several neurobiological and psychological mechanisms are involved in the placebo effect [4,5], which can even produce improvements in the relief of subjective symptoms such as pain, fatigue, and depression [6]. On the other hand, the nocebo effect can arise as a negative psychobiological response to a supposed treatment [7,8].

The placebo effect has been extensively studied in sport [9,10,11,12,13,14] with most studies conducted using deceptive ergogenic aids (e.g., caffeine, carbohydrates, or sodium bicarbonate) [3].

Caffeine is widely used as a deceptive substance because it is one of the most commonly employed dietary supplements as an ergogenic aid for improving sports performance [15]. Its ergogenic effects have also been reported across a variety of sports [16,17,18], where caffeine ingestion has been associated with improved performance. In endurance events such as running, cycling, rowing, and triathlon, caffeine has been used to produce a placebo effect due to its known ergogenic effects in these sports [19,20]. Thus, different authors have used caffeine as an element of suggestion for athletes in placebo studies [1,11,12,13]. During long duration exercise, where physiological requirements are predominantly aerobic, caffeine used as a placebo has been studied before. The time to complete a 4 km performance test was shorter in the caffeine reported/caffeine received and caffeine reported/placebo received conditions compared to the control condition (no intake), without differences between the two experimental conditions in time or RPE [12], demonstrating a placebo effect in recreational athletes. In middle-distance trained athletes, Hurst, Schipof-Godart [11] conducted a 1 km performance test with four conditions: caffeine reported/caffeine received, caffeine reported/placebo received, placebo reported/caffeine received, and placebo reported/placebo received. The time to complete 1 km was shorter in the caffeine reported/caffeine received and caffeine reported/placebo received conditions. It was also observed that participants started faster during the first half of the test, suggesting that the caffeine reported/placebo received condition exerted psychological effects and led to changes in the distribution of effort (pacing strategy). Finally, in a recent study [13], 13 trained long-distance runners performed a 6-min run test under two conditions: caffeine reported/placebo received and no intake (control). The total distance covered was measured and 400 m split times were used to analyze the pacing strategy selected by the runners. The results showed that placebo ingestion improved 6-min run performance compared to the control condition, without affecting pacing. Consequently, deceptive caffeine ingestion can improve performance at intensities close to maximal aerobic speed [13].

Therefore, caffeine-induced expectancy may be one of the mechanisms behind the ergogenic effect of this stimulant in endurance exercise [1,11,12,13]. According to the current scientific literature, the placebo effect could be considered an effective strategy for improving performance, as it poses minimal health risks to athletes and may reduce the side effects associated with other ergogenic aids, such as caffeine, which has been shown to cause increased nervousness and sleep disturbances, among other side effects [21,22,23].

Although the available evidence supports the existence of a placebo effect derived from caffeine expectancy in endurance performance, it has been obtained exclusively from studies using simulated competitions or single-effort time-trial protocols. To date, this effect has not been examined during regular interval training sessions, which constitute a core component of endurance training programs. If caffeine-induced expectancy improves endurance performance (as measured by time trials or time-to-exhaustion tests), the absolute speed achieved during an interval training session may also increase. Time sustained at a high fraction of maximal oxygen consumption (VO_2_max; e.g., ≥90%) has emerged as a key metric for evaluating the effectiveness of interval training protocols [24]. Since daily training largely determines athletes’ long-term performance progression, it remains unknown whether caffeine expectancy could similarly enhance performance under these ecologically relevant conditions and thereby contribute to overall competitive performance. Therefore, the present study aimed to analyze the potential placebo effect of the belief that one had ingested a moderate dose of caffeine on performance during an interval training running session in trained runners.

## 2. Materials and Methods

### 2.1. Experimental Design

A randomized, counter-balanced, repeated-measures experimental design was used to compare the effects of ingesting a placebo reported as caffeine (placebo condition) with a condition in which no substance was ingested (control condition) before an interval training session. The study was designed and reported following the CONSORT 2010 guidelines [25]; the completed checklist is available in Supplementary Table S1. Participants completed three interval training sessions in total: one familiarization session, followed by the placebo and control conditions, all separated by 7 days and administered in a computer-generated randomized, counter-balanced order to avoid any order effect. After participant enrolment, the principal investigator generated the computer-generated random allocation sequence, assigning participants in a 1:1 ratio to one of the two treatment sequences (placebo–control or control–placebo). The same investigator enrolled the participants and assigned them to one of the two treatment sequences. The allocation sequence was not disclosed to participants before assignment. Participant recruitment was conducted in March 2024. Experimental testing was subsequently scheduled over the following 12 months according to each participant’s availability, considering their competitive calendar and personal schedules. The familiarization session allowed participants to become accustomed to the interval training protocol, the outdoor track, and the equipment used, thereby minimizing any learning effect on the subsequent experimental sessions. Prior to the study, all participants were informed about the study protocols and potential risks involved and were invited to give written informed consent. The study was conducted in accordance with the principles of the Declaration of Helsinki, and the experimental protocols were approved by an ethics committee (ref. 28.1.2021CEI-UCJC). The study was retrospectively registered on the Open Science Framework (OSF: https://osf.io/; osf.io/vj4bx) on 30 January 2026. This trial was not registered prospectively because, at the time the study was planned, it did not meet the local definition of a clinical trial, as it did not investigate the clinical, pharmacological, or pharmacodynamic effects of a medicinal product. Accordingly, the study was not subject to mandatory trial registration under Spanish regulations [26].

### 2.2. Participants

An a priori power analysis (G*Power v3.1.9.7) indicated that nine participants were required to detect a placebo effect of caffeine (effect size = 1.15; two-tailed paired *t*-test; 1 − β = 0.80; α = 0.05), based on the placebo versus control comparison reported by Hurst et al. [11]. This calculation referred to the primary outcome (total time to complete the 5 × 1000-m session). Because the present study also included repeated physiological, perceptual, and kinematic measurements across five intervals per condition, five additional participants were recruited to increase the robustness of the repeated-measures analyses. Therefore, 14 trained male runners (mean ± SD, 25.93 ± 9.14 years of age; 67.93 ± 6.41 kg; 176.43 ± 5.33 cm; 9.64 ± 6.39 years of running experience, 59.29 ± 21.29 km of weekly training volume; 17:00 ± 01:45 personal best at 5 km) participated voluntarily in this study. The inclusion criteria were: (1) regularly performing endurance running training at least 3 days per week during the previous year; (2) holding a valid athletics federation license and regularly participating in official competitions throughout the year; (3) being low-to-mild habitual caffeine consumers, with a daily caffeine intake between 25 mg/day and 2.99 mg/kg/day [27]; and (4) having no physical limitations, musculoskeletal injuries, or respiratory or cardiovascular diseases preventing sports practice in the last six months.

### 2.3. Procedure

**Figure 1:**
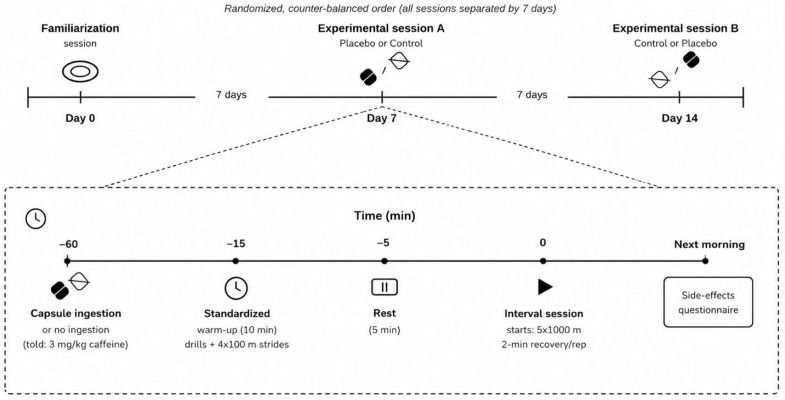
Schematic representation of the experimental protocol.

Participants were instructed to avoid caffeine intake and high-intensity exercise during the 48 h preceding the three training sessions. Twenty-four hours before each experimental test, participants were instructed to adopt similar sleep patterns, diet, and fluid intake regimens. All tests were conducted at the same time of day to avoid the effects of circadian rhythms [28] and under similar weather conditions (no rain and similar ambient temperature). Figure 1 schematically illustrates the experimental procedure.

Before each session, participants were informed about the ergogenic properties of caffeine and how previous studies had found benefits in endurance running performance with its use. Then, participants ingested a capsule containing a placebo substance (100 mg of corn flour) but were informed that they had received 3 mg·kg^−1^ caffeine 60 min before performing the interval training session for the experimental condition. After this, participants performed a standardized warm-up consisting of 10 min of easy running, mobility drills, running technique drills, and 4 × 100-m strides. Participants then rested for 5 min before the interval training session. The interval training session consisted of 5 × 1000 m with 2 min of recovery between repetitions, where they were instructed to perform each interval at the maximum sustainable intensity for that session (“isoeffort”) [29,30] in each condition. All sessions were conducted on a standard 400-m outdoor track (Mondo, Alba, Italy) by the World Athletics Federation’s standards. The times of each 1000-m interval (primary outcome) and each 200-m split time were recorded manually (a method recognized by World Athletics as official and acceptable for competition settings [31]) by two experienced researchers. Mean values of both times were used to analyze the pacing strategy adopted by each athlete. In addition, heart rate was recorded with an H9 pectoral band (Polar Electro Oy, Kempele, Finland) connected to a Garmin Forerunner 55 watch (Garmin, Olathe, KS, USA).

The main kinematic variables of the gait cycle (step frequency, vertical oscillation, and ground contact time) were measured for each 400-m split using an inertial measurement unit (Stryd power meter, Stryd Inc., Boulder, CO, USA) with a sampling frequency of 1000 Hz that has shown adequate validity and reliability compared to optical measurement devices for measuring spatiotemporal parameters [32]. These variables were selected because they provide objective information on running biomechanics and have previously been used to investigate the effects of caffeine expectancy on running performance and pacing strategy [13]. For each test, participants were instructed to wear the same shoes (checked by the researchers) due to the potential influence of advanced footwear technology on running performance, and similar clothing. Also, participants did not receive any information on split times or heart rate throughout the trial.

Finally, the morning after the training sessions, the participants answered a digital questionnaire on possible side effects (Google Forms), where they had to indicate on a dichotomous scale (yes/no) the presence or absence of each possible side effect, such as nervousness, digestive problems or difficulty sleeping, as well as rate on a scale from 1 to 10 the intensity of these effects. This questionnaire was previously used to assess the side effects of caffeine intake in sports [23].

### 2.4. Statistical Analysis

Data are presented as mean ± standard deviation (SD). Statistical analyses were performed using IBM SPSS Statistics version 29 (IBM Corp., Armonk, NY, USA), with statistical significance set at *p* < 0.05.

Performance, rating of perceived exertion (RPE), heart rate, and running biomechanics variables (stride frequency, ground contact time, vertical oscillation) were analysed using linear mixed-effects models fitted by restricted maximum likelihood (REML). For each dependent variable, condition (control vs. placebo), interval (1–5), and their interaction were included as fixed effects, whereas participant was included as a random intercept to account for repeated observations within individuals. Repeated measurements across the five intervals within each condition were modelled using a first-order autoregressive covariance structure. Alternative covariance structures were explored during model development, and the final structure was selected based on the lowest Akaike Information Criterion (AIC). Bonferroni-adjusted pairwise comparisons were performed when significant interaction effects were detected. For the main effect of condition, estimated marginal means (EMMs) and their corresponding 95% confidence intervals (95% CI) derived from the fitted models were reported (EMM [95% CI]).

Prior to the final analyses, treatment order (control–placebo vs. placebo–control) was evaluated as an additional fixed effect to assess a potential sequence effect. As treatment order was not statistically significant for any outcome variable, it was omitted from the final models to retain the most parsimonious model. Model assumptions were evaluated by visual inspection of residual plots and assessment of residual normality.

Pacing strategy was analysed using an additional linear mixed-effects model applied to the 200-m split times, incorporating split (200–1000 m) as an additional fixed factor together with all corresponding interaction terms. The same estimation method, random-effects structure, covariance structure, and post hoc procedures described above were applied.

A paired t-test was used to compare overall 5 × 1000-m performance (total time) between the placebo and control conditions. Ratings of side effects were compared using the Wilcoxon signed-rank test because these variables were measured on an ordinal scale and showed a high proportion of tied observations. Additionally, the average change in performance (total time) was standardized and expressed as a factor of small worthwhile change (SWC), a small, standardized effect based on Cohen’s effect size principle (0.2 × between-athletes standard deviation, SD) [33]. Finally, the effect size (ES) for the pairwise comparisons was calculated using Cohen’s *d*, interpreting it as <0.20 trivial, ≥0.20–0.59 small, ≥0.60–1.19 moderate, ≥1.20–1.99 large, and ≥2.00 very large according to the recommendations of Hopkins [34].

## 3. Results

**Figure 2:**
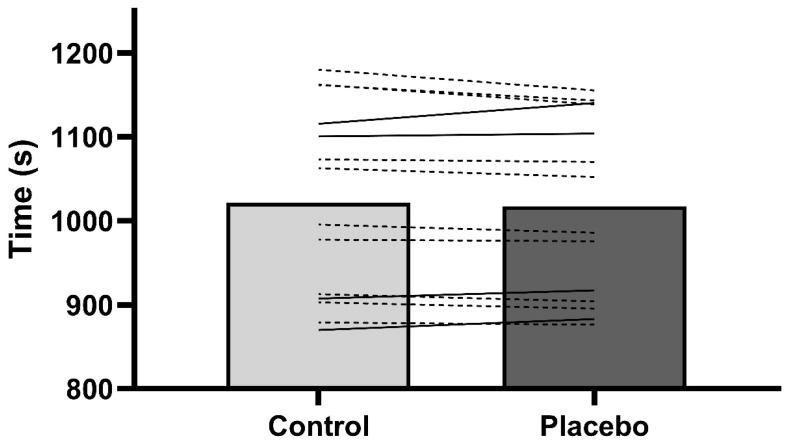
Accumulated time for 5 × 1000 m. The columns represent the mean values for each condition, and the lines represent individual data. The dashed lines indicate athletes who recorded a shorter time in the placebo condition, and the solid lines indicate athletes who recorded a shorter time in the control condition.

Seven participants were allocated to the placebo–control sequence and seven to the control–placebo sequence. All participants completed both periods and were included in the analyses. No significant improvement in overall performance was observed across the 5 × 1000-m training session under the placebo condition (1021.5 ± 114.0 s control vs. 1017.2 ± 109.1 s placebo; *p* = 0.270, Cohen’s d = 0.31, small effect). Despite 9 of the 14 participants recording faster total times with placebo (Figure 2), the magnitude and consistency of the individual responses were insufficient to produce a significant overall effect.

Moreover, Table 1 shows performance times recorded during each 1000-m interval under the placebo and control conditions. A significant condition × interval interaction was observed (F = 3.32, *p* = 0.014), together with a significant main effect of interval (F = 4.01, *p* = 0.005), whereas no main effect of condition was found (EMM [95% CI]: 204.3 [191.5, 217.1] vs. 203.4 [190.6, 216.3]; F = 0.31, *p* = 0.585). Pairwise comparisons revealed no significant differences between the placebo and control conditions at any individual 1000-m interval (all *p* > 0.05). In the pacing analysis, the condition × interval × split interaction was not significant (F = 0.40, *p* = 0.984). Although a significant condition × split interaction was detected (F = 2.54, *p* = 0.039), pairwise comparisons revealed no significant differences between the placebo and control conditions at any individual 200-m split (all *p* > 0.05).

Both HR and RPE exhibited a similar response pattern. No main effect of condition was found for HR (175.1 [170.5, 179.7] vs. 175.8 [171.2, 80.3]; F = 0.374, *p* = 0.552) or RPE (8.57 [8.22, 8.92] vs. 8.57 [8.22, 8.92]; F < 0.001, *p* = 1.000). Conversely, a significant main effect of interval was observed for both variables (HR: F = 40.376, *p* < 0.001; RPE: F = 51.982, *p* < 0.001), reflecting the expected increase in physiological and perceptual demands across the training session. No significant condition × interval interaction was detected for either HR (F = 0.201, *p* = 0.937) or RPE (F = 1.152, *p* = 0.339), indicating that placebo ingestion did not modify the evolution of HR or perceived exertion across the five intervals.

Similarly, the running kinematics variables exhibited a consistent response pattern. No main effect of condition (all *p* > 0.05) was observed for step frequency (179.7 [175.0, 184.3] vs. 178.9 [174.2, 83.5]), vertical oscillation (8.24 [7.81, 8.68] vs. 8.32 [7.89, 8.76]), or ground contact time (181.7 [171.8, 191.5] vs. 182.3 [172.5, 192.2]). In contrast, a significant main effect of interval was detected for all three variables (all *p* < 0.05), indicating progressive changes in running mechanics throughout the interval session. However, no significant condition × interval interactions were found (all *p* > 0.05), suggesting that placebo ingestion did not alter the temporal evolution of these biomechanical variables across the five intervals.

Finally, in the perceived side effects questionnaire (Table 2), we observed significant differences in the item related to activeness, where 8 out of 14 participants reported feeling more activeness in the placebo condition compared to the control condition (4.14 ± 2.69 and 1.71 ± 1.33 for placebo and control conditions, respectively; *p* = 0.017; moderate Cohen’s *d* = 0.85). Significant differences were also found in the items related to increased urine production and insomnia (moderate Cohen’s *d* ≈ 0.6) in the placebo condition compared to the control condition.

## 4. Discussion

The aim of this study was to analyze the potential placebo effect associated with caffeine ingestion before an interval training session (5 × 1000 m with 2 min of rest) in trained endurance runners. The main findings of this study were: (a) the total time to complete the training session was similar between conditions; (b) there were no differences in the average heart rate for each 1000-m interval between conditions; (c) no significant differences were observed in the measured kinematic variables; and (d) placebo ingestion increased perceived activeness and the perception of selected caffeine-related side effects.

Total time to complete the 5 × 1000 m was similar between control and placebo conditions. These findings contrast with previous studies in which a placebo effect was observed, leading to an improvement in performance in 1 km TT [11], 4 km TT [12], and 6-min TT [13] tests. Although no significant differences were found in our study, the total time required to complete the training session under the placebo condition was 4.27 s lower, representing a 0.42% improvement in performance compared to the control condition. However, this improvement is noticeably smaller than those reported in previous studies. A possible explanation for these results could be the nature of the effort itself, as our study used an interval training session rather than a performance test. Regarding individual responses, 9 out of 14 participants had a lower total time (exceeding the SWC) to complete the training session under the placebo condition (range of 2–25 s), while 4 participants ran faster in the control condition (improvements ranging from 3 to 25 s), and one participant showed virtually no difference between conditions. Although this pattern may suggest a degree of individual variability in the response to the placebo condition, such differences could also simply reflect normal day-to-day fluctuations in performance, which are common among endurance athletes even in the absence of any intervention. Given the small sample size, the current data do not allow firm conclusions to be drawn regarding the existence of distinct responder and non-responder profiles, and this observation should therefore be interpreted with caution.

In competitions, athletes must regulate their speed to reach the endpoint of the race in the fastest possible time [35]. However, during interval training sessions, the athlete must be able to self-regulate effort according to the demands and duration of the activity along with the recovery period [35]. In the present study, participants were instructed to complete each interval at the highest sustainable intensity while maintaining the same recovery duration between repetitions. Therefore, the only factor expected to influence pacing strategy was the belief that they had consumed caffeine. Although the linear mixed model revealed a significant condition × interval interaction, together with a main effect of interval, Bonferroni-adjusted pairwise comparisons showed no significant differences between the placebo and control conditions at any of the five 1000-m intervals. This suggests that the significant interaction reflected an overall pattern across the repeated measurements rather than robust differences at any specific interval. Accordingly, caffeine expectancy did not meaningfully modify pacing behavior during the interval training session. Consistent with these findings, the 200-m split analysis showed no significant condition × interval × split interaction. Although a significant condition × split interaction was observed, pairwise comparisons also revealed no significant differences between the placebo and control conditions at any individual 200-m split. Taken together, these results indicate that caffeine expectancy did not modify either the between-interval or within-interval pacing pattern during the 5 × 1000-m training session. This finding is noteworthy, as the limited previous literature suggests that participants start faster when they believe they have ingested caffeine [12,13]; however, this response likely depends on the type of test performed (e.g., interval vs. time trial), participants’ subjective perceptions of caffeine’s potential benefits, and the pacing strategy they adopt [36].

Regarding the average heart rate for each 1000-m interval, no significant differences were observed between conditions. Given that no relevant changes were observed in the total time during the session (and therefore, no increase in running speed [external load]), it is logical that no changes were found in the internal load (HR). In addition, it is well-established that environmental conditions affect heart rate [37], mainly through cardiovascular drift caused by body water losses during exercise in hot conditions. For that reason, these environmental conditions were controlled, being similar for each athlete on both testing days.

RPE was similar in both conditions, indicating no placebo effect in this perceived variable according to other previous studies. Regarding the perception of subjective effort, as observed in previous studies [11,13], no significant differences were found between the two trials (control 8.57 ± 0.53 vs. placebo 8.57 ± 0.63; *p* = 1.000). This variable may be influenced by psychological factors (e.g., a potential sensation of feeling better with placebo ingestion) and by the participants’ running experience, as it is a subjective measure and therefore more challenging to quantify accurately.

Concerning running kinematic variables, vertical oscillation, step frequency, and ground contact time were analyzed using an inertial measurement unit. These variables have only been previously used as performance indicators in running, since there is only one study [13] that has employed them to assess whether well-trained runners altered their running kinematics when investigating the placebo effect of the belief that they had ingested caffeine. As observed in this previous study, athletes did not modify their running technique (at least in terms of the variables measured by the inertial measurement unit) in the placebo condition compared to the control condition, with values being similar in both conditions. A possible explanation is that kinematic changes depend largely on speed changes [38], and the slight increase in speed observed in the lower total average time to cover the 5 × 1000 m training session in the placebo condition might not have been sufficient to produce significant running kinematic changes.

Finally, to analyze potential side effects, participants completed a questionnaire on the morning following each experimental session. A total of 57.14% (8 participants) reported feeling more energetic when they believed they had consumed caffeine, consistent with previous findings [13], with significantly higher ratings than in the control condition. Significant differences were also observed for increased urine production and sleep disturbances, with higher ratings in the placebo condition than in the control condition. In contrast, no significant differences were found for the remaining side effects, including irritability, gastrointestinal discomfort, and sleep quality. Although these findings suggest that caffeine expectancy may increase the perception of some expected caffeine-related side effects, the overall magnitude of these differences was small. Therefore, these results should be interpreted within the exploratory context of the present study and should not be considered evidence of a consistent or generalizable effect of caffeine expectancy on side effects or sports performance.

This study has several limitations. First, no female athletes volunteered to participate, resulting in an exclusively male sample. This limits the generalizability of the findings, as placebo responses may be influenced by biological sex. However, the available evidence on sex-related differences in placebo effects remains limited and heterogeneous, particularly in sports settings, with most data originating from clinical research [39,40]. Second, comparing the placebo condition with a no-ingestion control, rather than with an inert capsule without expectancy manipulation, does not allow full isolation of caffeine-related expectancy from other non-specific effects of capsule ingestion itself. Third, we did not include a manipulation check to confirm that participants genuinely believed they had ingested caffeine, nor did we statistically examine whether habitual caffeine consumption influenced the magnitude of the placebo response. This is an important limitation, as previous evidence suggests that the magnitude of placebo-induced performance improvements depends more on participants’ expectancy of having consumed caffeine than on the experimental condition itself [1]. Future studies should include a post-trial manipulation check to disentangle these effects. Finally, although all sessions were conducted under similar weather conditions (i.e., no rain and comparable ambient temperature), scheduling them at comparable times of day and within the same season to minimize environmental variability, temperature and humidity were not formally recorded or strictly standardized, and minor day-to-day fluctuations cannot be entirely ruled out as potential confounding factors. Nevertheless, previous evidence suggests that meteorological factors, particularly ambient temperature and wind, are more likely to influence endurance running performance when conditions differ substantially or fall outside the optimal range for performance [41]. Given the apparently similar conditions across testing sessions, it is unlikely that small variations in weather meaningfully affected the present findings. In addition, the exercise protocol consisted of five 1000-m intervals separated by 2-min recovery periods, which may have facilitated partial heat dissipation and limited the progressive accumulation of thermal strain, potentially further reducing the influence of modest environmental differences on performance. However, this interpretation should be considered with caution, as the effects of small variations in meteorological conditions during intermittent endurance running protocols have not been directly investigated.

## 5. Conclusions

In conclusion, caffeine expectancy did not significantly affect overall interval-running performance, pacing strategy, physiological responses, or running biomechanics in trained runners. Likewise, perceived exertion remained unchanged between conditions. However, participants reported greater feelings of activeness and a higher perception of selected caffeine-related side effects under the placebo condition. Overall, these findings suggest that caffeine expectancy had no influence on objective performance-related measures during interval-training and only a small influence on self-perceived measures after interval-training sessions.

## Figures and Tables

**Table 1:** Performance, heart rate, rating of perceived exertion, and running kinematics variables across the five intervals. Values are presented as mean ± SD. The last column shows the estimated marginal mean (EMM) difference (Control − Placebo) and corresponding 95% confidence interval (95% CI) obtained from the linear mixed-effects models.

Variable	Interval	Control	Placebo	*p*	EMM Difference (Control − Placebo) [95% CI]
Interval performance (s)	1st	202.42 ± 20.99	205.65 ± 21.42	0.109	−3.23 [−7.22, 0.77]
	2nd	204.97 ± 22.15	202.87 ± 20.65	0.290	2.11 [−1.89, 6.10]
	3rd	204.75 ± 23.41	203.60 ± 21.77	0.561	1.15 [−2.84, 5.14]
	4th	206.12 ± 25.67	204.13 ± 22.84	0.317	1.99 [−2.00, 5.98]
	5th	203.23 ± 23.81	200.98 ± 23.24	0.259	2.25 [−1.74, 6.24]
Heart rate (bpm)	1st	171.1 ± 6.7	171.3 ± 7.6	0.861	−0.2 [−2.7, 2.3]
	2nd	174.5 ± 7.9	175.2 ± 7.9	0.560	−0.7 [−3.2, 1.8]
	3rd	175.9 ± 8.0	176.4 ± 8.2	0.641	−0.5 [−3.1, 1.9]
	4th	176.6 ± 8.6	177.4 ± 8.7	0.560	−0.7 [−3.2, 1.8]
	5th	177.5 ± 9.0	178.5 ± 9.2	0.417	−1.0 [−3.5, 1.5]
RPE (u.a.)	1st	7.64 ± 0.93	7.43 ± 1.09	0.324	0.21 [−0.22, 0.65]
	2nd	8.14 ± 0.54	7.93 ± 0.73	0.324	0.21 [−0.22, 0.65]
	3rd	8.43 ± 0.65	8.64 ± 0.75	0.324	−0.21 [−0.65, 0.22]
	4th	9.07 ± 0.62	9.14 ± 0.66	0.741	−0.07 [−0.50, 0.36]
	5th	9.57 ± 0.76	9.71 ± 0.47	0.510	−0.14 [−0.58, 0.29]
Step frequency (steps·min^−1^)	1st	179.57 ± 7.64	177.64 ± 7.48	0.114	1.93 [−0.50, 4.36]
	2nd	179.50 ± 7.29	178.57 ± 8.13	0.437	0.93 [−1.50, 3.36]
	3rd	179.57 ± 7.83	178.43 ± 8.83	0.340	1.14 [−1.29, 3.57]
	4th	179.64 ± 8.06	179.29 ± 8.45	0.763	0.36 [−2.07, 2.79]
	5th	180.14 ± 8.01	180.43 ± 10.01	0.810	−0.29 [−2.71, 2.14]
Contact time (ms)	1st	178.50 ± 15.01	181.29 ± 17.49	0.163	−2.79 [−6.76, 1.19]
	2nd	180.93 ± 15.61	181.50 ± 17.36	0.772	−0.57 [−4.54, 3.40]
	3rd	182.57 ± 16.22	183.36 ± 17.92	0.690	−0.79 [−4.76, 3.19]
	4th	183.79 ± 18.31	183.57 ± 19.07	0.913	0.21 [−3.76, 4.19]
	5th	182.57 ± 17.86	182.00 ± 19.36	0.772	0.57 [−3.40, 4.54]
Vertical oscillation (cm)	1st	8.35 ± 0.74	8.47 ± 0.67	0.322	−0.13 [−0.39, 0.13]
	2nd	8.28 ± 0.72	8.39 ± 0.80	0.379	−0.11 [−0.37, 0.15]
	3rd	8.23 ± 0.74	8.36 ± 0.87	0.328	−0.13 [−0.38, 0.13]
	4th	8.18 ± 0.79	8.23 ± 0.84	0.652	−0.06 [−0.32, 0.20]
	5th	8.17 ± 0.74	8.16 ± 0.93	0.932	0.01 [−0.25, 0.27]

**Table 2:** Side effects experienced by participants after both tests.

	Control	Placebo	*p*	Cohen’s *d*
Nervousness	1.57 ± 1.16	2.50 ± 1.70	0.127	0.46
Activeness	1.71 ± 1.33	4.14 ± 2.69	0.017 *	0.85
Irritable	1.14 ± 0.36	1.07 ± 0.27	1.000	0.27
Muscular pain	1.79 ± 1.22	1.57 ± 0.94	0.605	0.17
Headache	1.07 ± 0.27	1.79 ± 1.89	0.181	0.38
Gastrointestinal discomfort	1.14 ± 0.54	2.00 ± 2.08	0.223	0.39
Increase urine production	1.07 ± 0.27	2.21 ± 1.81	0.035 *	0.62
Insomnia	1.14 ± 0.54	1.64 ± 1.08	0.048 *	0.59
Sleep quality	7.86 ± 2.14	8.07 ± 1.44	0.916	0.11

Note: asterisks (*) indicate statistical differences (*p* < 0.05).

## Data Availability

The original contributions presented in this study are included in the article/Supplementary Material. Further inquiries can be directed to the corresponding author.

## Supplementary Materials

The following supporting information can be downloaded at: https://www.mdpi.com/article/10.3390/nu18152492/s1, Table S1: CONSORT checklist of information to include when reporting randomised crossover trials.
